# Gastro-protective effect of *Biebersteinia multifida *root hydro-methanolic extract in rats with ethanol-induced peptic ulcer 

**Published:** 2019

**Authors:** Mahdi Raeesi, Narges Eskandari-Roozbahani, Tahoora Shomali

**Affiliations:** 1 *Division of Pharmacology and Toxicology, Department of Basic Sciences, School of Veterinary Medicine, Shiraz University, Shiraz, Iran.*

**Keywords:** Biebersteinia multifida, Peptic ulcer, Rat, Antioxidant, Anti-inflammatory

## Abstract

**Objective:**

*Biebersteinia multifida* is one of the native plants of Iran and its root is used in folk medicine. This study aimed to evaluate the gastro-protective effect of the hydro-methanolic extract of this plant's roots against ethanol-induced gastric ulcer in rats.

**Materials and Methods:**

The following five groups of seven rats were included in this study: control (C), gastric ulcer (GU), control omeprazole (CO) and two treatment groups (the latter 3 groups were rats with gastric ulcer that orally received omeprazole, 20 mg/kg, or the root extract at 150 and 300 mg/kg (BM 150 and BM 300, respectively) 1 hour before ulcer induction). One hour after ulcer induction, blood sampling was performed and after sacrificing animals, the stomachs were immediately removed. Gastric mucosal injury was studied grossly to determine the number and area of gastric ulcers. The level of nitric oxide (NO) and total antioxidant capacity (TAC) in gastric mucosa as well as serum TNF-α were determined.

**Results:**

In GU group, severe mucosal injuries were observed (p<0.0001 as compared to C group). The lesions in CO and treatment groups were much milder than GU group by regarding ulcer area and number (p<0.001 for all cases). In treated (BM 150 and BM 300) groups, the gastric mucosal TAC and NO level were significantly higher than GU group (p<0.05 for all cases). Serum TNF-α level was not significantly different between GU and other groups.

**Conclusion:**

*B. multifida *possesses gastro-protective effects against ethanol-induced ulcer model; this effect is at least partly related to plant’s antioxidant and NO production accelerating properties.

## Introduction

A common gastrointestinal disease is peptic ulcer that primarily affects the stomach and the duodenum (Konturek et al., 2005 ▶). A wide range of factors may cause gastric ulcer including excessive production of gastric acid or pepsin, insufficient mucosal defense, reﬂux of bile and pancreatic juice into the stomach, *Helicobacter pylori*, smoking, alcohol consumption, non-steroidal anti-inﬂammatory drugs use and psychosocial stress (Richardson, 1990 ▶; Everhart et al., 1998 ▶). Although ethanol consumption is a risk factor for gastric ulcer, the magnitude of its effect depends on the level of consumption and is most damaging in very heavy drinkers (Razvodovsky, 2006 ▶), however, there is a weak association between alcohol consumption and ulcers in moderate drinkers (Chou, 1994 ▶).

Different mechanisms are proposed for ethanol induced-stomach ulcers including oxidative stress (Repetto and Llesuy, 2002 ▶), microcirculation disruption (Hernandez-Munoz et al., 2000 ▶), infiltration of neutrophils, secretion of inflammatory mediators (De Souza et al., 2011 ▶), loss of the protective layer of gastric epithelial cells and subsequently increased vulnerability to HCl and pepsin secreted into gastric lumen (Oates et al., 1988 ▶).

Ethanol-induced gastric ulcer model is different from other models of gastric ulcer that majorly rely on gastric acid secretion (Brazozowski et al., 1998 ▶). In fact, this model is commonly recommended for studying factors which have cytoprotective and/or antioxidant properties (Adinorty et al., 2013).

Drugs which are routinely used to treat gastric ulcer cause side effects; moreover, long-term treatment is usually needed and above all, no complete recovery may be achieved. Therefore, alternative and/or adjunct approaches are demanded. Herbal medicines have shown a diverse range of different mechanisms for their effects on peptic ulcers including acceleration of mucous cell proliferation, antioxidant properties, and reduction of gastric acid secretion (Bi et al., 2014 ▶). These agents especially those with a history of use in traditional medicine might be beneficial for treatment of and protection against peptic ulcers usually with proper safety and cost-effectiveness (Bi et al., 2014 ▶). 

*Biebersteinia* is a genus of plants in the flowering plant order Sapindales (Christenhusz and Byng, 2016 ▶). They occur from the East Mediterranean to West Siberia and Central Asia (Muellner, 2011 ▶) with *Biebersteinia multifida *DC (BM) as the native species of Iran. In folk medicine, the ointment made of the tuberous root of this plant has been used for curing muscle and skeletal disorders and bone fractures (Amin, 1991 ▶; Farsam et al., 2000 ▶; Amirghofran, 2010 ▶). Besides, BM has been used for the treatment of nocturia in children (Aboutorabi, 2001 ▶). Alkaloids, flavonoids, and polysaccharides are the basic constituents of BM (Kurbanov and Zharekeev, 1974 ▶; Omurkamzinova et al., 1991; Arifkhodzhaev and Rakhimov, 1994 ▶). Flavonoids including 7-glucosides of apigenin, luteolin, and tricetin, as well as the 7-rutinoside of apigenin and luteolin, contribute to the antioxidant and anti-hemolytic activities of BM (Greenham et al., 2001; Omurkamzinova et al., 1991; Nabavi et al., 2010 ▶). Other main components of BM include (E)-nerolidol, phytol, 6, 10, 14-trimethyl-2-pentadecanone and hexadecanoic acid (Javidnia et al., 2010). In different studies, diverse beneficial effects of the plant like anti-inflammatory and analgesic effects (Farsam et al., 2002 ▶) as well as anti-bacterial (Godrati et al., 2012 ▶), anti-hemolytic and anti-oxidant activities (Nabavi et al., 2010 ▶), have been confirmed. It has also been found effective against some psychological disorders (Monsef-Esfahani et al., 2013 ▶).

Considering the successful use of BM in traditional medicine, and antioxidant and anti-inflammatory effects of this plant, we were persuaded to evaluate the gastro-protective effect of BM's extract against ethanol-induced gastric ulcer *in vivo.*

## Materials and Methods

**Preparing BM root extract**

Taxonomical identification of fresh plant was done by the research center of natural products health (NPH), North Khorasan University of Medical Sciences (Iran), after collecting BM in April 2016 from the mountains of Khorasan Province, northeastern Iran. To prepare the hydro-methanolic extract, dried root powder of BM (100 g) was percolated with methanol 70% (1.0 L) in a sterile environment for 5 days. After filtration and removing the solvent under vacuum at 40°C, 10 g of extract was yielded. In order to prepare the appropriate dose of the extract, distilled water was used as diluent. Doses (150 and 300 mg/kg) were chosen based on our pilot tests.

**Animals and study design**

Thirty-five male Wistar rats (200-250 g) were used in this study, they were maintained on a standard diet, water *ad libitum*, ambient temperature around 23°C, and 12/12 light/dark cycle and were left for a week for adaptation. The food was withdrawn 24 hours before the experiment but water was freely available. Rats were randomly allocated into 5 groups of 7 animals each as follows. 

1: Control group (C), normal rats that received no treatment during the experiment; 2: Gastric ulcer (GU) group, gastric ulcer was induced in this group and the animals were treated with distilled water, 3: Control omeprazole group (CO), gastric ulcer was induced in this group and the animals received omeprazole (Abidi Pharmaceutical Co., Iran), 20 mg/kg (Segawa et al., 1987 ▶) in distilled water, and 4 and 5: BM treatment groups, gastric ulcer was induced in these groups and the animals received extract of BM at 150 and 300 mg/kg, respectively. One hour before induction of peptic ulcer (by oral administration of 4 ml/kg of 75% ethanol (Singh et al., 2007) to groups 2-5), different agents were administered orally. 

One hour after ulcer induction, blood sampling was done under diethyl ether anesthesia via cardiocentesis. After that, animals were sacrificed by deepening anesthesia and the stomachs were removed quickly. After opening the stomach from its greater curvature, it was rinsed with normal saline. Photographs were taken and glandular part of the stomachs was assessed for ulcer formation. Mucosal damages in glandular part of the stomach were determined as hemorrhagic streaks or linear breaks (erosions) in the mucosal surface. 

All procedures were done in accordance with institutional ethical guidelines for use of experimental animals and were consistent with European convention for the protection of vertebrate animals used for experimental and other scientific purposes.

**Determination of number and area of peptic ulcers**

The number of ulcers was enumerated and planimetric method was used for calculating the ulcer area (Shomali et al., 2014 ▶) using Axio Vision Software. Then, stomachs were immediately transferred to a -70°C freezer.

**Analysis of the gastric tissue nitric oxide (NO) content and total antioxidant capacity (TAC)**

After weighing the glandular part of each stomach, samples were homogenized in cold phosphate buffered solution pH 7.4 (100 mg/ml); then, the homogenized samples were centrifuged (at 4°C and 9500 rpm for 5 min) and supernatant was removed and used as the sample for analyzing NO content (Pan et al; 2005) and total antioxidant capacity (TAC) was measured by colorimetric methods (Shomali et al., 2014 ▶). The assays were done according to guidelines of the manufacturer of the kits prepared by Biocore Diagnostik (ZellBio), Germany.

**Determination of serum tumor necrosis factor-α (TNF-α) level**

For measuring TNF-α, blood samples were used. The samples were centrifuged (3000 rpm for 10 min) and sera were harvested and kept at −70°C until analysis (Du et al., 2013 ▶). TNF-α was measured by an ELISA kit (Biorbyt’s rat TNF-α ELISA kit, UK) based on the sandwich ELISA method and according to the manufacturer instructions. The absorbance of the specimens was determined using an ELISA reader at 450 nm and the levels of TNF-α were expressed as pg/ml.

**Statistical Analysis**

Data are expressed as mean±SD and analyzed statistically by the analysis of variance (one way ANOVA) method. Differences among groups were investigated using Tukey's multiple comparison tests and p<0.05 was considered the level of significance. 

## Results

**The number and area of ulcers**

**Figure 1:**
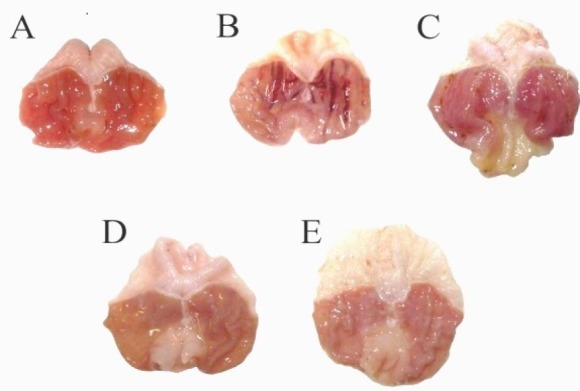
Effect of hydro-methanolic extract of *Biebersteinia multifida* root on gastric ulcers in the glandular part of the stomach from rats in different groups. The severity of lesions was most prominent in gastric ulcer group, while rats that received omeprazole or *B. multifida* showed milder changes

Severe hyperemia and hemorrhage were observed in stomachs of GU group. The severity of lesions was much lower in groups treated with omeprazole and BM extract at both doses (Figure 1). 

A: Control; B: Gastric ulcer; C: Control omeprazole (omeprazole 20 mg/kg); D and E: *Biebersteinia multifida* 150 and 300 mg/kg, respectively.

**Figure 2:**
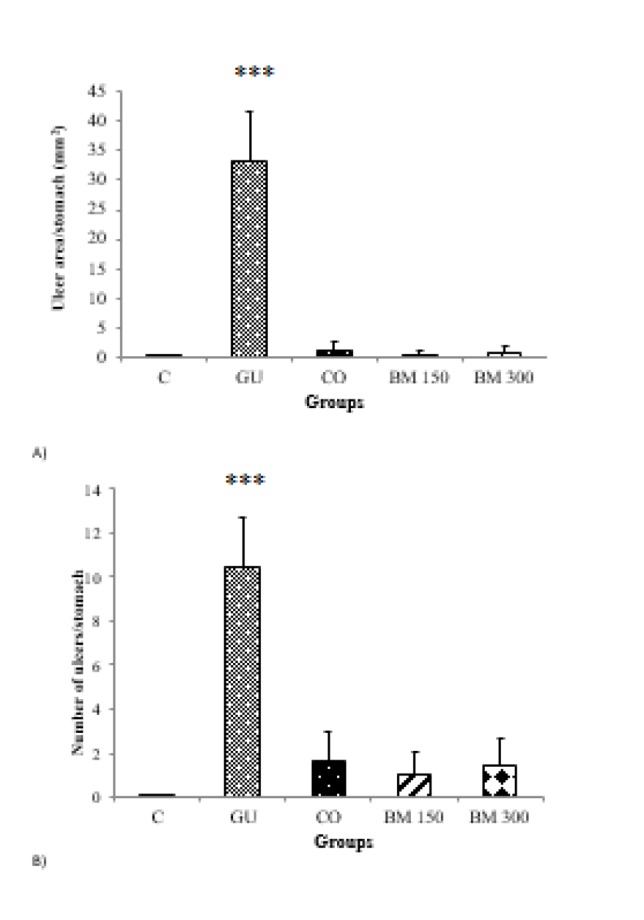
Effect of hydro-methanolic extract of *Biebersteinia multifida* root on the ulcer area (A) and number (B) /stomach in rats. Data are presented as mean±SD. ***p<0.001 as compared to other groups. C: Control; GU: Gastric ulcer; CO: Control omeprazole (omeprazole 20 mg/kg, orally); BM 150 and BM 300 mg/kg: *B. multifida* 150 and 300 mg/kg by oral administration

Data on total ulcer area and number in stomachs of rats in different groups are presented in Figures 2A and 2B; a significant difference was found in this regard between GU group and other groups (p<0.001 for all cases). Ulcer area and number in treatment groups and CO group were statistically similar to that of the C group (p>0.05) (Figure 2 A and 2 B).

No significant difference was observed between BM 150 and BM 300 groups for both parameters (p>0.05).

**Mucosal TAC and NO content and serum TNF-α level**

**Figure 3:**
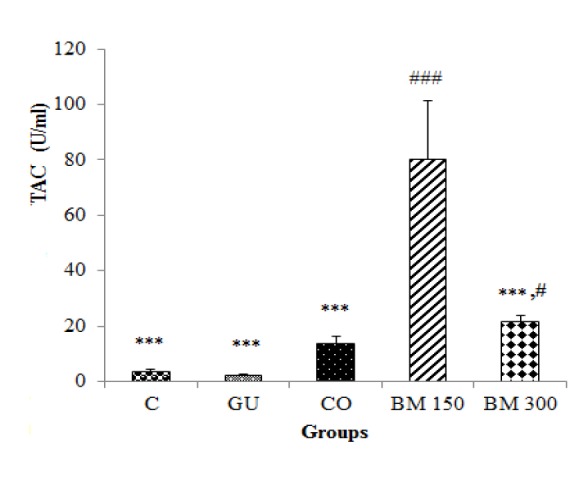
Effect of hydro-methanolic extract of *Biebersteinia multifida* root on total antioxidant capacity (TAC) of gastric mucosa in gastric ulcer induced by ethanol in rats. Data are presented as mean±SD. #p<0.05, ### p<0.001 as compared to C and GU groups; ***p<0.001 as compared to BM 150 group. C: Control; GU: Gastric ulcer; CO: Control Omeprazole (omeprazole 20 mg/kg, orally); BM 150 and BM 300 mg/kg: *B. multifida* 150 and 300 mg/kg by oral administration

Data showed that mucosal TAC in rats which were treated by BM 150 and BM 300 mg/kg was significantly higher than that of GU (p<0.001 and p<0.05, respectively) and C groups (p<0.001 and p<0.05, respectively). Mucosal TAC in BM 150 group was also significantly higher than BM 300 and CO groups (p<0.001 for both cases). No significant change was observed in mucosal TAC of GU and CO groups as compared to C group (p>0.05) (Figure 3).

**Figure 4:**
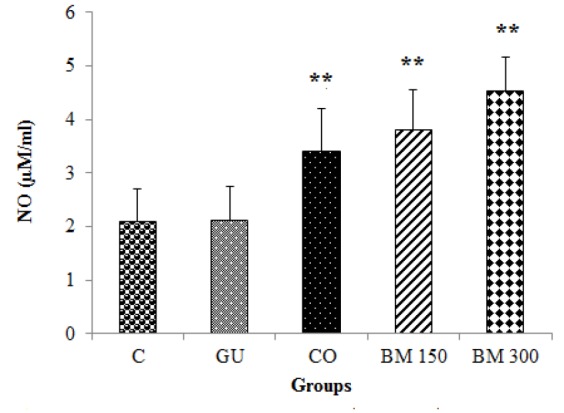
Effects of hydro-methanolic extract of *Biebersteinia multifida* root on nitric oxide (NO) content of gastric mucosa in gastric ulcer induced by ethanol in rats. Data are presented as mean±SD. **p<0.01 as compared to C and GU groups. C: Control; GU: Gastric ulcer; CO: Control Omeprazole (omeprazole 20 mg/kg, orally); BM 150 and BM 300 mg/kg: *B. multifida* 150 and 300 mg/kg by oral administration

In relation to mucosal NO content, in rats which were treated by BM (150 and 300 mg/kg) and in CO group, NO content was significantly higher as compared to GU and C groups (p<0.01 for all cases). There was no significant difference between BM 300 and BM 150 group (p>0.05). This parameter was significantly higher in BM 300 as compared to CO group (p<0.05) (Figure 4).

**Figure 5:**
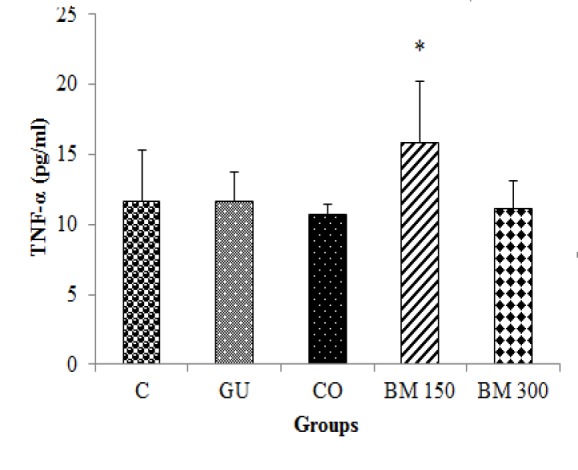
Effect of hydro-methanolic extract of *Biebersteinia multifida* root on serum TNF-α level in gastric ulcer induced by ethanol in rats. Data are presented as mean±SD. * p<0.05 as compared to CO group C: Control; GU: Gastric ulcer; CO: Control Omeprazole (omeprazole 20 mg/kg, orally); BM 150 and BM 300 mg/kg: *B. multifida* 150 and 300 mg/kg by oral administration

Serum level of TNF-α was statistically higher in rats treated with BM 150 mg/kg as compared to CO group (p<0.05). Other groups showed statistically similar levels of serum TNF-α in a way that, induction of ulcer in GU group was not associated with a significant change in serum TNF-α levels as compared to the C group (Figure 5).

## Discussion

We examined the protective effect of BM extract against ethanol-induced gastric ulcer in rats. Since the ethanol-induced gastric ulcer is more dependent on disturbances in cytoprotective mechanisms, secretion of inflammatory mediators and antioxidant properties (De Souza et al., 2011 ▶; Repetto and Llesuy, 2002 ▶; Hernandez-Munoz et al., 2000 ▶), for studying the protective effect of BM on this gastric ulcer model, we examined some relative parameters which included total antioxidant capacity and mucosal NO content as well as serum TNF-α level. 

As stated above, the results of the total ulcer area and number in stomachs of rats in different groups, revealed that severe mucosal injury was present in GU group but the lesions in CO and treatment groups were very mild as compared to GU group.

Data showed that remission of gastric ulcers by both doses of BM was accompanied by higher mucosal TAC and NO content. 

Phytochemical studies have revealed that BM contains neutral polysaccharides described as glucans A, B, and C (Arifkhodzhaev et al., 1985 ▶; Arifkhodzhaev and Rakhimov, 1993 ▶, 1994) and alkaloids (Kurbanov and Zharekeev, 1974 ▶). It is believed that BM's biological activities are related to the presence of phenols, flavonoids and alkaloid components of the plant.

It was suggested that disturbances in the mucus-bicarbonate barrier and cell membrane rupture in the wall of blood vessels in ethanol-induced gastric ulcer are presumably due to lipid peroxidation, formation of free radicals and intracellular oxidative stress. Therefore, oxidative stress, as a major player in this regard, has a pivotal role in the creation of necrotic injuries (Sannomiya et al., 2005 ▶). Consistently, in our study, the appreciable preventive effect and high mucosal TAC were observed in BM extract-treated groups.

In previous studies, Greenham et al. (2001) and Omurkamzinova et al. (1991) characterized some of BM's compounds including polysaccharides, peptides, alkaloids such as vasicinone, and flavonoids like 7-glucosides of apigenin, luteolin, and tricetin, as well as 7-rutinoside of apigenin and luteolin (Greenham et al., 2001; Omurkamzinova et al., 1991). Nabavi et al. (2010) ▶ revealed the anti-hemolytic and antioxidant effects of the BM extract. It is well established that some of the pharmacological properties of BM like antioxidant and antihemolytic activities, are because of the presence of vasicinone (Monsef-Esfahani et al., 2013 ▶; Greenham et al., 2001). Considering our findings about reducing the number and extent of ulcers by BM, it can be concluded that these effects may be related to antioxidant effects of this extract, as shown by higher mucosal TAC.

Nitric oxide is known for regulating gastric blood flow. In the stomach, NO helps to maintain gastric mucosal health and can, therefore, be a protective factor against ethanol damage (TeppermanandSoper, 1994 ▶). Nitric oxide is produced by nitric oxide synthase (NOS) enzymes including gastric endothelial NOS (eNOS) and inducible NOS (iNOS). Among them, eNOS activity is a pivotal factor in the protection of gastric mucosa while iNOS can participate in ulcer formation through the production of peroxide free radicals (Cho, 2001 ▶). In a study done in mice, it was shown that 1 hour after peptic ulcer induction, the expression of eNOS and production of NO in gastric mucosa is high, but after 3 and 6 hours, the expression of iNOS increases. In the present study, for determination of NO content, sampling of gastric tissue was done 1 hour after ulcer induction, hereupon; the increased level of NO due to BM extract is most possibly related to eNOS activity (Pan et al., 2005 ▶). 

In our study, omeprazole administration also resulted in significant NO elevation and also showed a tendency to increase TAC. 

Although omeprazole is routinely used in gastric ulcer because of its ability in suppressing gastric acid secretion by inhibiting the proton pumps (Shin and Sachs, 2008 ▶), a previous study showed that the acid-reducing effect of omeprazole contributes little to its protective effect in ethanol-induced gastric ulcer (Le et al., 2001 ▶). In fact, a major part of the gastro-protective effect of omeprazole is relevant to its potent antioxidant activity (Biswas et al., 2003 ▶; Shomali et al., 2014 ▶).

Moreover, in a study by Le et al. (2001) ▶ on rats with ethanol-induced gastric ulcer, it was observed that omeprazole can exert important protection against gastric mucosal lesion through NO, which is also in agreement with our results.

TNF-α is a pro-inflammatory cytokine that has an important role in the maintenance and regulation of the severity of peptic ulcers (Choi et al., 2010 ▶) as well as gastric mucosal apoptosis (Nakashita et al., 2013 ▶). Du et al. (2013) ▶ reported a significant increase in TNF-α concentration of gastric mucosa of rats with ethanol-induced gastric ulcer after 1 hour which was reduced in rats treated with *Veronica strumaxillare* (Du et al., 2013 ▶). In 2014, Li et al showed that 4 hours after ethanol administration in mice, TNF-α level in gastric tissue and serum was significantly increased which was attenuated by chelerythrine alkaloid (Li et al., 2014 ▶). As stated above, we evaluated serum levels of TNF-α 1 hour after ulcer induction. This can explain why there was no significant change in this parameter in rats of GU group as compared to C group. Concerning the effect of BM extract on TNF-α, the only significant change was the appreciable increase of this parameter in the BM 150 mg/kg-treated rats as compared to CO group. A previous investigation reported that administration of BM aqueous extract at 500 mg/kg for 8 days to rats with acetic acid-induced ulcerative colitis, results in a significant decrease in intestinal TNF-α levels (Keshavarzi et al., 2018 ▶). These controversies may be related to different factors including the type of the extract, dose, duration of administration, site of evaluation, etc.

Proton pump inhibitors like omeprazole were shown to affect the production of pro-inflammatory cytokines (nuclear factor-κB (NF-κB) and interleukin-8 (IL-8) (Kedika et al., 2009 ▶). We did not observe a significant difference in TNF-α level between the CO group and C or GU groups. In 2006, Handa et al. showed that the effect of proton pump inhibitors on pro-inflammatory cytokines is related to gastric ulcer model, e.g. induction of peptic ulcer by *Helicobacter pylori*, which induces a significant increase in cytokines (Handa et al., 2006 ▶). Therefore, one of the most important reasons for the inconsistency between these results and the results of our study, can be related to the model of gastric ulcer induction. Consistently, in a similar study by Shomali et al. (2016) ▶, similar results were obtained regarding the effect of omeprazole on TNF-α.

*B. multifida *root hydro-methanolic possesses gastro-protective properties against ethanol-induced peptic ulcer model that is, at least partly, related to its antioxidant properties and NO accelerating effects. Further studies are necessary to clarify other possible mechanisms.
